# Designing a monitoring program for aflatoxin B1 in feed products using machine learning

**DOI:** 10.1038/s41538-022-00154-2

**Published:** 2022-09-01

**Authors:** X. Wang, Y. Bouzembrak, A. G. J. M. Oude Lansink, H. J. van der Fels-Klerx

**Affiliations:** 1 Business Economics, Wageningen University, Wageningen, The Netherlands; 2 Wageningen Food Safety Research, Wageningen, The Netherlands

**Keywords:** Economics, Chemical safety

## Abstract

Agricultural commodities used for feed and food production are frequently contaminated with mycotoxins, such as Aflatoxin B1 (AFB1). In Europe, both the government and companies have monitoring programs in place for the presence of AFB1. With limited resources and following risk-based monitoring as prescribed in EU Regulation 2017/625, these monitoring programs focus on batches with the highest probability of being contaminated. This study explored the use of machine learning algorithms (ML) to design risk-based monitoring programs for AFB1 in feed products, considering both monitoring cost and model performance. Historical monitoring data for the presence of AFB1 in feed products (2005–2018; 5605 records in total) were used. Four different ML algorithms, including Decision tree, Logistic regression, Support vector machine and Extreme gradient boosting (XGB), were applied and compared to predict the high-risk feed batches to be considered for further AFB1 sampling and analysis. The monitoring cost included the cost of: sampling and analysis, disease burden, storage, and of recalling and destroying contaminated feed batches. The ML algorithms were able to predict the high-risk batches, with an AUC, recall, and accuracy higher than 0.8, 0.6, and 0.9, respectively. The XGB algorithm outperformed the other three investigated ML. Its incorporation would result into up to 96% reduction in monitoring cost in 2016–2018, as compared to the official monitoring program. The proposed approach for designing risk based monitoring programs can support authorities and industries to reduce the monitoring cost for other food safety hazards as well.

## Introduction

The Food and Agriculture Organization (FAO) estimated that 25% of global food crops are contaminated with mycotoxins^1^. Recently, the prevalence of the mycotoxins detected in food and feed crops was reported to be up to 60–80%^2^. Aflatoxins are the major ones among all mycotoxins and produced by *Aspergillus* spp. upon and after infection of crops. They are genotoxic and carcinogenic to animals and humans^3–6^. From the different aflatoxins, aflatoxin B1 (AFB1) is found most often in agricultural commodities used for feed and food production, like peanuts, maize, and rice, in particular in tropical and subtropical regions. AFB1 causes adverse health effects, such as liver cancer^7^. When AFB1 is present in the feed of dairy cows and digested, it converts to aflatoxin M1 (AFM1), which is excreted in the milk. Long-term exposure to aflatoxin M1, for example through consumption of contaminated dairy products, can lead to DNA damage, cancer or immunosuppression in humans, and large doses can even lead to acute poisoning^7,8^. To keep animal and human exposure of AFB1 as low as possible, many countries worldwide have set maximum (legal) limits for its presence in feed and food^9,10^. Next to the impact on human and animal health, the presence of AFB1 in feed or its ingredients above the maximum legal limits can lead to major economic losses, for example due to extra testing, recall, downgrading or destruction^11,12^.

In Europe, the European Commission (EC) No. 574/2011 has set maximum limits for the presence of AFB1 in feed^13^ and has defined sampling and analysis (S&A) procedures for AFB1 monitoring in Regulation (EC) No 401/2006 of 23 February 2006^14^. In addition, procedures for official food safety control have been set by Regulation (EC) No 882/2004 and Regulation (EU) 2017/625, stating that Member States should establish and implement control programs for contaminants in feed and food materials and derived products. The latter regulation implies risk-based control programs, i.e., feed batches that present a high-risk of AFB1 non-compliance-, should be collected and analyzed^15,16^.

Food safety authorities and companies generally want to ensure a low probability of contamination in feed, while limiting the resources for AFB1 monitoring^17^. Thus, the optimal monitoring (sampling and analysis) program should be risk-based, that is, focused on monitoring only high-risk batches to limit monitoring resources, or to increase the probability of detection of high-risk batches with a given set of resources.

Previous studies have explored the two above-mentioned aspects related to AFB1 monitoring, mainly aimed at the potential for risk-based monitoring in the EU and the cost-effectiveness of S&A procedures. Van der Fels-Klerx, et al.^18^ used descriptive statistical analyses—focusing on the occurrence of AFB1 in different feed products - to prioritize feed products for AFB1 monitoring. Furthermore, Van der Fels-Klerx, et al.^19^ developed a model to prioritize individual feed ingredients for AFB1 monitoring, according to the impact of their contamination on animal and human health. Focker, et al.^17^ estimated the cost-effectiveness of several S&A methods for AFB1 monitoring in the maize chain. Wang et al.^20^ optimized the S&A monitoring plan for multiple chemicals, including dioxins and aflatoxins, along the dairy chain to reduce potential public health impacts. The models for risk-based monitoring are highly valuable for prioritizing feed or food products for AFB1 monitoring. The cost-effectiveness S&A approach can help to reduce the cost while meeting the required monitoring effectiveness, or to improve the monitoring effectiveness with given resources.

However, to date, risk-based monitoring and cost-effectiveness monitoring have been studied separately. The combination of these two approaches could help in designing a risk-based monitoring program while limiting the monitoring resources.

To date, only a few studies have investigated machine learning (ML) methods for risk-based monitoring. ML has been widely used in other fields such as food science and medical science^21–26^ suggesting ML modeling might also be helpful for addressing the task of AFB1 prediction^27^. What’s more, most of these studies apply ML accuracy-based criteria (such as accuracy, recall, and area under the receiver operating characteristic curve (AUC)) to evaluate the ML model, while few studies use non-accuracy-based criteria (such as monitoring cost). For many commercial applications of ML models, accuracy is not the primary goal of the model (Kuhn & Johnson, 2013). In the case of AFB1 monitoring, the primary goal of the ML model might be to reduce monitoring costs while still meeting accuracy-based predictive criteria. Predictive accuracy is important, it describes how well the model predicts the real life situations (i.e., correct or not), while the criterium—of quantifying the consequences (i.e., associated cost) of correct and incorrect model predictions—monitoring costs in this case—should also be considered. For example, for the correct prediction of contaminated batches, there is a quantifiable benefit to catching these “flagged” feed batches. Likewise, for incorrect prediction of uncontaminated batches, there are cost of the recall and health losses due to “unflagged” contaminated batches entering the food supply chain and finally being used for human consumption. The objective of this study was to explore the potential of machine learning algorithms (ML) to design a risk-based food safety monitoring program for AFB1 in feed products considering both monitoring cost and model performance. This study contributes to the literature by being combining risk-based and cost-effectiveness monitoring. It is also innovative since it integrates an economic dimension (i.e., cost-effectiveness) with accuracy-based criteria, in the evaluation of the ML model performance.

## Results

### ML module

Table 1 presents a few examples of the ML model validation results, including predicted status and actual status of a feed batch, of whether or not the presence of AFB1 in the feed batch was compliant to the EC legal limit, given the input information related to the feed batches. In the first row, for example, given the month number is 3, the product is groundnuts, the product group is pulses, the origin country is China, and the analysis country is the Netherlands, the ML model predicted the feed batch as high-risk of AFB1 non-compliance; in line with the actual status. In the second row, the ML model predicted a high-risk as well, which was not in line with the actual status of the feed batch.

**Table 1:** Examples of ML model validation results.

Month	Product	Product subgroup	Product group	Country of origin	Country of analysis	Above or below legal limits	
						Actual result	Predicted result
3	Groundnuts/Peanuts	Groundnut/Peanut	Pulses	CN	NL	1	1
3	Groundnuts/Peanuts	Groundnut/Peanut	Pulses	BR	NL	0	1
12	Soya Beans, Extracted	Soya Bean	Oil Bearing Seeds	AR	AR	0	0

Results consist information (month, product name, product subgroup/group, country of origin, country of analysis) and predicted status and actual status of a feed batch (whether or not the presence of AFB1 in the feed batch was compliant to the EC legal limit).

**Fig. 1:**
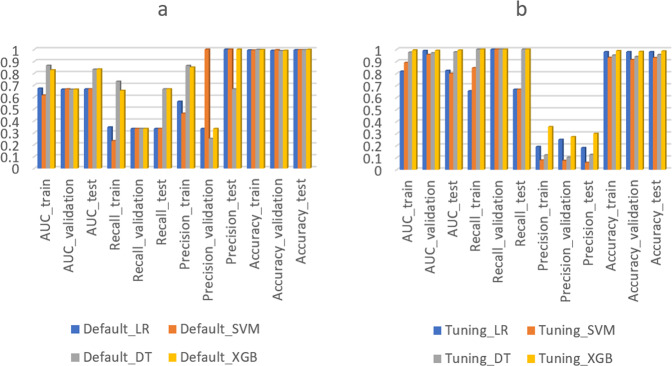
Model performance for four different algorithms (including Extreme gradient boosting (XGB), Decision tree (DT), Logistic regression (LR), and Support vector machine (SVM)), with default parameter settings (a) and with tuned parameter settings (b). Default_LR, Default_SVM, Default_DT, and Default_XGB represent ML algorithms using default parameter settings. Tuning_LR, Tuning_SVM, Tuning_DT, and Tuning_XGB represent ML algorithms using tuned parameter settings. AUC_train, AUC_validation, and AUC_test, represent AUC score on train, validation, and test dataset. Recall_train, Recall_validation, and Recall_test, represent recall score on train, validation, and test dataset. Precision_train, Precision_validation, and Precision_test, represent precision score on train, validation, and test dataset. Accuracy_train, Accuracy_validation, and Accuracy_test, represent accuracy score on train, validation, and test dataset.

Figure 1a shows the model performance of four different ML algorithms using default parameter settings. The average AUC, recall, precision, and accuracy were around 0.7, 0.3, 0.4, and 1, respectively. The recall score did not meet the model requirement of 0.8, meaning the model could not identify at least 80% of the contaminated feed batches. Figure 1b shows the model performance of ML algorithms with tuned parameter settings. The AUC, recall, precision, and accuracy were around 0.9, 1, 0.1, and 0.9, respectively. Compared to the default settings, the recall score increased, meaning the ML model could identify a higher percentage of the non-compliant batches, but the precision score and accuracy decreased, meaning the ML model misclassified more compliant batches as non-compliant. Hence, a trade-off occurs between precision and recall score when changing the parameter settings^28^. The XGB algorithm (with tuned parameters) outperformed the other three ML algorithms, showing the highest AUC (0.99), recall (1), precision (0.3), and accuracy (0.98) (Fig. 1b). Since the threshold for identifying non-compliant samples was set at 80%, LR algorithms resulting in a recall score of around 0.7 were excluded.

### Cost of the monitoring program

**Fig. 2:**
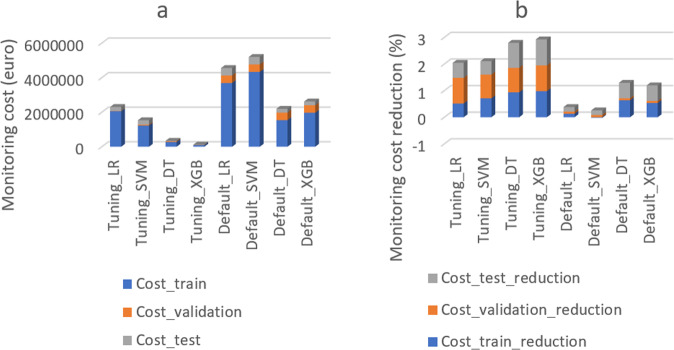
The cost of the designed monitoring program (a) and the percentage of cost reduction (b) using four different machine learning algorithms (including Extreme gradient boosting (XGB), Decision tree (DT), Logistic regression (LR), and Support vector machine (SVM)), each with default and tuned model parameters. Default_LR, Default_SVM, Default_DT, and Default_XGB represent ML algorithms using default parameter settings. Tuning_LR, Tuning_SVM, Tuning_DT, and Tuning_XGB represent ML algorithms using tuned parameter settings. AUC_train, AUC_validation, and AUC_test, represent AUC score on train, validation, and test dataset. Cost_train, Cost_validation, and Cost_test, represent monitoring cost on train, validation, and test dataset. Cost_train_reduction, Cost_validation_reduction, and Cost_test_reduction, represent monitoring cost reduction on train, validation, and test dataset.

Figure 2 shows the cost of the designed monitoring program—using different ML algorithms—calculated by formulas (1) to (4). The XGB algorithm (with tuned parameters) resulted in the lowest monitoring cost (121,488 EUR) and the highest monitoring cost reduction (97%) as compared to the other three ML algorithms. Thus, the XGB algorithm was the best performing ML algorithm considering both monitoring cost and model performance (in section of ML module). Figure 2 shows the percentage of monitoring cost reduction using different ML algorithms. Applying the DT and XGB algorithm for the designed monitoring plan resulted in lower monitoring cost (cost reduction of around 270% for the train, test, and validation dataset in total) as compared to the current monitoring plan. In contrast, applying the LR and SVM algorithm resulted in higher monitoring cost. This indicates that comparing and choosing the best performing algorithm for the predictive model is necessary, especially when the goal of the predictive model is not only prediction accuracy but also its practical application. Thus adding non-accuracy-based criteria (monitoring cost in this case) is recommended when designing a predictive model.

### Application of the monitoring plan using 2016–2018 dataset

**Fig. 3:**
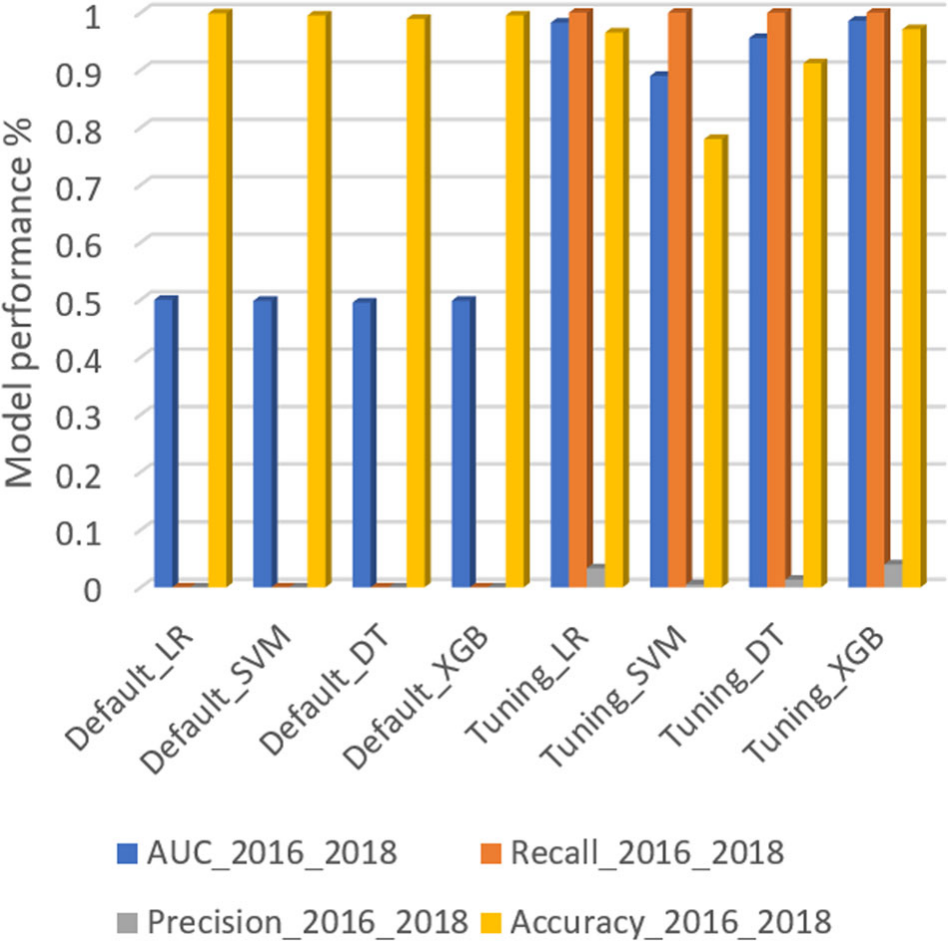
Model performance for four different machine learning algorithms (including Extreme gradient boosting (XGB), Decision tree (DT), Logistic regression (LR), and Support vector machine (SVM)), with default and tuned parameters settings, using the 2016–2018 dataset. Default_LR, Default_SVM, Default_DT, and Default_XGB represent ML algorithms using default parameter settings. Tuning_LR, Tuning_SVM, Tuning_DT, and Tuning_XGB represent ML algorithms using tuned parameter settings. AUC_2016_2018 represent AUC score on 2016–2018 dataset. Recall_2016_2018 represent recall score on 2016–2018 dataset. Precision_2016_2018 represent precision score on 2016–2018 dataset. Accuracy_2016_2018 represent accuracy score on 2016–2018 dataset.

Figure 3 shows the model performance of different ML algorithms for predicting high-risk batches using the dataset of 2016–2018 (external validation results). The XGB model consistently showed the best performance using internal model validation (Fig. 1b) and external validation (Fig. 3). This outcome confirmed that the selection of the XGB model (tuned) for the designed monitoring was stable and reliable with an AUC of 0.98, recall of 1, precision of 0.04, and accuracy of 0.97. Although the LR algorithm resulted in a similar performance (Fig. 3), it had low recall and AUC scores (Fig. 1b), which means the LR algorithm performed well for the 2016–2018 data by chance.

Table 2 presents a few examples of external validation results of the best performing ML model (XGB) using the 2016–2018 dataset. Only some of the predicted high-risk batches are presented in this Table since these are the batches of highest concern to stakeholders. For the feed material of groundnuts (with an EC legal limit of 0.02 mg/kg), feed batches with the features of analysis month of 1, 3, 7, and 9; country of origin China, India, and Argentina, and country of analysis the Netherlands, were predicted to be at high-risk of AFB1 non-compliance. For the feed material of maize (with a legal limit of 0.02 mg/kg), feed batches with the analysis month of 2 and 3, and country of origin Ukrain and country of analysis the Netherlands were predicted as high-risk as well. Out of the total of 239 feed batches, 238 batches were compliant and one batch was non-compliant. Result shows that 16 batches predicted to be high-risk AFB1 batches. From these, only one batch was in line with the actual status, i.e., detected as being non-compliant.

**Table 2:** Examples of the predicted high-risk batches using the best performing ML algorithm for 2016–2018.

Month	Product	Product subgroup	Product group	Country of origin	Country of analysis^a^	Above or below legal limits	
						Actual result	Predicted result
1	Groundnuts/Peanuts	Groundnut/Peanut	Pulses	IN	NL	0	1
7	Groundnuts/Peanuts	Groundnut/Peanut	Pulses	CN	NL	0	1
9	Groundnuts/Peanuts	Groundnut/Peanut	Pulses	AR	NL	0	1
3	Groundnuts/Peanuts	Groundnut/Peanut	Pulses	CN	NL	1	1
2	Maize	Maize	Grains	UA	NL	0	1
3	Maize	Maize	Grains	UA	NL	0	1

^a^The country of analysis is not always NL, but the batches analyzed in NL were being predicted as high-risk batches.

Table 3 presents the top ten features that had the highest feature importance. Among all the feed materials, rice bran, groundnuts, barley, and coconut were the ones with the highest feature importance. Among all the countries of origin, feed materials coming from Hungary and the United States of America had the highest feature importance.

**Table 3:** Ten most important features of the selected ML model, with the features ranked based on their respective score, and the score representing the importance of an feature contributing to the construction of the XGB model.

Features	Score of feature importance
**Product: Rice**	0.080
**Product group: Groundnut/Peanut**	0.067
**Country of analysis: EG**	0.044
**Country of origin: HU**	0.036
**Country of origin: US**	0.035
**Product group: Palm kernel**	0.034
**Product: Barley**	0.032
**Country of analysis: NL**	0.032
**Product group: Coconut**	0.031

#### Monitoring cost

Table 4 presents the estimated cost of the official AFB1 monitoring program and the designed monitoring program (using the XGB algorithm) for feed products in 2016–2018. Among all the 841 feed batches, 25 batches were predicted to be high-risk batches of being non-compliant (TP + FP), and 816 batches were predicted to be compliant (TN + FN). In the official control program, a total of 841 feed batches were flagged for S&A. Out of these, only one batch had an AFB1 concentration exceeding the legal limit. The total cost for the monitoring program was estimated at 925100 euros. In the designed optimal monitoring program, using the best performing ML algorithm, only 25 high-risk feed batches were flagged for S&A, at a cost of 32300 euros, achieving a monitoring cost reduction of 96% (at least 82% per year).

**Table 4:** The estimated cost of the official monitoring program and the designed monitoring program for AFB1 in feed in 2016–2018.

Monitoring program of 2016–2018	Total number of samples	ML model prediction result		Total cost (euro)	Calculation
Designed	841	TP	1	32300	Eqs. (1–4)
		FP	24		
		TN	816		
		FN^a^	0		
Official^b^	841	NAN^b^	NAN	925100	Eq. (5)

^a^TP represents an actual non-compliant batch that was predicted as non-compliant. FP represents a compliant batch that was predicted to be non-compliant, TN represents an actual compliant batch that was predicted as compliant. FN represents an actual non-compliant batch that was predicted as compliant.

^b^No predictive model was performed.

## Discussion

This study explored the potential of using ML algorithms for the design of a risk-based monitoring program for AFB1 in feed, considering the monitoring cost and model performance as evaluation criteria. To our knowledge, to date, monitoring cost has not yet been used as non-accuracy-based criteria in the evaluation of the model performance for several ML algorithms. Another innovative aspect of this study is that it uses the prediction result of ML to design a risk-based monitoring program for monitoring AFB1 in feed in a cost-effective way. This study showed that ML algorithms were able to predict, with a high prediction performance, which feed batches were most likely to be contaminated with AFB1. The designed monitoring program resulted in a large reduction of the monitoring cost by focusing S&A on high-risk batches only: cost were estimated to be up to 96% lower than the costs of the official monitoring program in 2016–2018. These saved resources could be applied for additional random testing of feed batches to identify the AFB1 prevalence or, given related data are available, to monitor other food safety hazards in various foods.

The finding that ML algorithms are able to predict the high-risk batches is in line with earlier studies of Bouzembrak and van der Fels-Klerx (2017) and Bouzembrak and Marvin (2019). These studies used Bayesian network modeling to predict whether various food safety hazards had high probabilities to be present in the particular feed or food product. ML modeling approaches can learn these patterns from historical food safety monitoring data to identify food safety risks. Our finding is also consistent with an earlier study that applied an ML approach (deep neural network), using weather and cropping system factors as input variables, to predict whether maize is contaminated with AFB1 and fumonisin^27^. In each of these three studies, one ML algorithm was applied, and only accuracy-based criteria were used to evaluate model performance. Kuhn and Johnson^29^ states that non-accuracy-based criteria is necessary to take into account for many commercial applications of ML models, because accuracy is not the primary goal of the model. Our research compared four different ML algorithms, and used both non-accuracy-based criteria (monitoring cost) and accuracy-based criteria (AUC, recall, and accuracy) for model evaluation. In our case, XGB outperformed the other algorithms, but this may be different with other datasets involving different costs. Thus, it remains necessary to compare various ML algorithms to select the ML algorithm with the best performance when designing a predictive model, as also concluded by Wang et al.^26^.

In addition, our approach use the prediction result of ML to design a risk-based monitoring program for monitoring AFB1 in feed in a cost-effective way. In contrast to Focker et al.^17^, our study focused on predicting high-risk batches (applying the S&A strategy for one batch in line with Regulation (EU) No. 401/2006), whereas Focker et al.^17^ focused on designing a cost-effective S&A strategy for one batch of feed material (maize in their case) to reduce the monitoring cost. To design a full monitoring program and to further reduce costs, both approaches should be combined. That is, first the high-risk batches should be predicted (to determine which batches should be monitored), and then the S&A strategy of Focker et al.^17^ for one batch should be applied (to determine how many samples to collect from one batch and which analytical method to use). Our approach also builds upon the work of Wang et al.^20^, who considered the reduction of disease burden when designing a model for feed industry to optimize the number of samples for monitoring aflatoxins. Their study estimated the impact of AFB1 contaminated feed batches on human health by estimating the loss of quality and quantity of life due to exposure to AFB1 via dairy milk consumption. Based on their research, our study further estimated the cost related to the loss of quality and quantity of life due to contaminated feed.

The monitoring cost was estimated from the perspective of feed factory, and the monitoring cost included in this study consisted of the costs of S&A for the tested feed batches, as well as the recall-related costs and the human disease burden costs of contaminated feed batches. Although there is no official regulation that states that the disease burden should be considered by feed industry, the European commission (EC) has set legal maximum limits for the presence of chemicals in food and animal feed products to protect human health via exposure to food safety hazards via food consumption. Since feed industries need to comply to this regulation, reducing the disease burden is implicitly considered. We assumed that the monitoring program was applied at a certain control point in the feed supply chain, being the moment that feed materials move from the trader to the feed company. This means the program was focused on monitoring feed material rather than monitoring compound feed. In practice, this control point at entry of the feed production is the most important one. In addition, we assumed a batch size of 100 tonne for one batch of feed materials. According to Regulation (EU) No 401/2006, the large batch should be subdivided into sub-batches (EU, 2010b) with weights in the range of 50–500 tonnes. Our assumed volume for one batch is within the range of such a sub-batch, and represents a regular batch size in the Netherlands^17^. Following Eqs. (1)–(5), changing the assumed weight per batch will change the monitoring cost, but will not affect the percentage of monitoring cost reduction. Furthermore, when having the S&A in a batch of 100 tonnes unprocessed feed materials, the measurement uncertainty and analytical variance were not considered in this study for sake of simplicity. In order to decide whether the batch was compliant with Regulation (EC) No. 181/2006, the result given by the analytical method, or the mean of the results in case of analysing multiple aliquots, was considered. Regulation No. 401/2006 suggests to take into account the measurement uncertainty when deciding if a batch is compliant or not. If the result is above the limit but the limit is within the uncertainty range, or if the mean result is below the limit and the limit is within the uncertainty range, the batch can be accepted (EU, 2010b). The variance of a sampling and analysis procedure was calculated as the sum of the variance due to the sample collection, the sample preparation, and the analysis of the sample (Whitaker, 2003). These two factors could slightly affect the test result of the sample/batch when the AFB1 concentration in feed is close to the legal limit (Whitaker, 2003).

We designed an risk based monitoring program for feed industry, but this monitoring program can also be applied by food safety authorities as long as the recall-related cost are not taken into account. If the monitoring program designed in the current study would be applied in practice, fewer batches would be collected and analyzed for the presence of AFB1. This would generate fewer historical monitoring records over time, in particular fewer records of compliant feed material batches. If the designed model would be applied going forward, each time using the latest historical monitoring results, the prediction performance of the ML model would decrease over time. The reason is in that only the predicted non-compliant batches are collected and analyzed for the presence of AFB1 (have S&A), and only these analytical results are stored in the historical monitoring dataset. In the short run (next few years), the effect on the ML model’s performance is expected to be small, since the historical monitoring data are usually highly unbalanced between compliant records and non-compliant records, i.e., the data contain a small number of non-compliant records and a large number of compliant records. In the long run, this could results into biased model performance due to fewer data used for modeling when no additional samples are collected. It is therefore recommended to add random sampling to the designed monitoring plan. That means samples are not only collected from the predicted non-compliant batches (TP + FP), but also additional samples are randomly collected from the predicted compliant batches (TN + FN). In this way, the annual monitoring data set could be more balanced.

The ML module was designed based on factors that can influence the presence of AFB1 and compliance to legal limits. Other factors such as weather conditions and agronomy might also influence the presence of AFB1 in feed materials (Camardo Leggieri et al.^27^; Kos et al.^30^; Leggieri et al.^31^; Munkvold^32^; Palumbo et al.^33^; Van der Fels-Klerx et al.^18^). For example, the probability of AFB1 contamination of a particular feed material from one exporting country might be affected by weather conditions (e.g., drought and/or flood) in that country, that vary over the years. A next research step could be to extend AFB1 monitoring models with data on weather conditions and possibly also other factors.

In conclusion, this study explored the potential of machine learning (ML) algorithms in designing a risk-based monitoring program for AFB1 contamination in feed materials, considering both monitoring cost and model performance. Our results showed that ML algorithms were able to predict the high-risk batches, given batch related conditions. The XGB algorithm outperformed the other ML algorithms under evaluation (Decision tree, Logistic regression, Support vector machine) based on a combination of non-accuracy-based (monitoring cost) and performance-based evaluation criteria (accuracy, recall, AUC). The findings of this study provide insights into the use of non-accuracy-based criteria to evaluate ML model performance in real-life applications. The study showed the designed monitoring program could greatly reduce AFB1 monitoring cost. Based on the official monitoring program in the Netherlands in the year 2016–2018, the monitoring cost could be reduced by up to 96% (at least 82% per year). Authorities and industries could use the designed monitoring program to focus S&A on the high-risk feed batches and reduce the monitoring cost.

## Method

### AFB1 monitoring

**Fig. 4:**
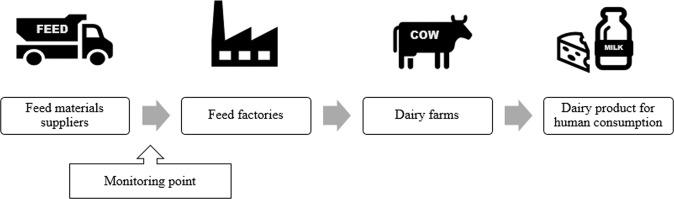
A simplified feed supply chain. The monitoring point along the feed supply chain for AFB1 was set prior to feed material entry feed factories.

A simplified feed supply chain was assumed consisting of the following stages (Fig. 4): feed materials are sold by the trader to the feed factories. Feed factories produce compound feed by mixing and processing different raw materials. The compound feed enters the dairy farms, where it is consumed by dairy cows that produce dairy milk. After processing, the milk is sold as the final product for human consumption. In accordance to practice, the monitoring point for AFB1 was set prior to feed material entry the feed factories, as the presence of AFB1 in feed products is to a large extent determined by its presence in raw materials^17^.

A risk-based AFB1 monitoring program was designed combining a machine learning (ML) module and an economic module. The ML module was used to predict high-risk feed batches, i.e., with a presence of AFB1 exceeding EU maximum legal limits, as set by European Commission (EC) No. 574/2011. The economic module aimed to estimate the cost of the monitoring program from the perspective of the feed industry. These two modules are described below.

### Data

The risk-based monitoring program was designed based on of historical monitoring data from the official AFB1 control program (4492 records) and private industry monitoring (1113 records) in the period 2005–2018 (5605 records in total). Most of the monitoring data represented the test results of S&A in the period 2005–2018 following Regulation (EC) No 401/2006. All samples were independent samples and analyzed once for the presence of AfB1. The monitoring records were retrieved from the Quality of Agricultural Products (KAP) database.

Table 5 presents some example records from the dataset with the information related to sample ID, date of sampling, product name, product subgroup, product group, hazard, country of origin, country of analysis, and the determined concentration of AFB1. We assumed that the monitoring information contained in one record represented the information of one feed batch, i.e., 5003 records represented 5003 batches.

**Table 5:** Example records from the dataset for the AFB1 monitoring program. Records consist the information related to sample ID, date of sampling, product name, product subgroup/group, specific food safety hazard, country of origin, country of analysis, and the determined concentration of one specific hazard.

Id	Date sampling	Product name	Product subgroup	Product group	Hazard	Country of origin	Country of analysis^a^	Concentration
2953	8/3/2005	Sunflower Seed	Sunflower	Oil Bearing Seeds	Aflatoxin B1	AR	NL	0.004 mg/kg
35792	4/21/2008	Maize	Maize	Grains	Aflatoxin B1	BR	NL	0.008 mg/kg

^a^Feed batches were analyzed in NL and other countries.

The monitoring data were used in both the ML and the economic module. Data from the years 2005 to 2015 were used to train and test the ML algorithms to predict the high-risk batches and to estimate the monitoring cost. In this step, the ML algorithm with the best prediction performance and lowest monitoring cost was selected for the design of the monitoring program. Data from the year 2016–2018 were used to test the designed monitoring program, and to compare the costs of the current and the newly designed monitoring programs.

### Machine learning module

The ML module was used to predict which feed batches had a high-risk of AFB1 non-compliance to EC legal limits using different ML algorithms. The predictive ability was defined as the effectiveness of the ML module to identify non-compliant feed batches. A minimal threshold of 80% was set for the effectiveness, meaning at least 80% of the non-compliant feed batches, i.e., with an AFB1 concentration above the respective ML, should be identified. This means the recall score of the ML model should be higher than 80%. In this AFB1 monitoring program, due to the low number of uncompliant samples (33 in total in the dataset), the value of monitoring effectiveness is set at 80%. This value can be set higher in different monitoring cases.

**Fig. 5:**
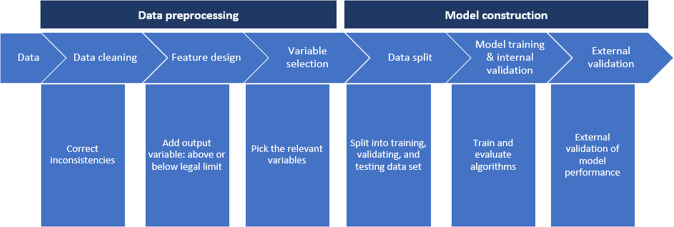
The development of the machine learning module, consisting of a data pre-processing part and a model construction part.

In the ML module, four algorithms were applied: Extreme gradient boosting (XGB), Decision tree (DT), Logistic regression (LR), and Support vector machine (SVM). A detailed explanation of these algorithms can be found in Géron^34^. The different types of information related to feed material batches (e.g., country of origin, country of analysis, product group, and month of sampling) were incorporated as model input variables, and compliance with the legislation limit (yes/no) was used as the output variable. The prediction results of the ML algorithms included the number of true positive (TP), true negative (TN), false positive (FP), and false negative (FN) batches. TP represents an actual non-compliant batch that was predicted as non-compliant. FP represents a compliant batch that was predicted to be non-compliant, TN represents an actual compliant batch that was predicted as compliant. FN represents an actual non-compliant batch that was predicted as compliant. Evaluation metrics for model performance, including AUC, recall, precision, and accuracy, were used to evaluate the predictive performance of the four different ML algorithms. AUC computes the area under the receiver operating characteristic (ROC) curve. ROC curve, is a graphical plot that illustrates the performance of a binary classifier system. It is created by plotting the fraction of true positives out of the positives (true positive rate) vs. the fraction of false positives out of the negatives (false positive rate), at various threshold settings. The recall is the ratio TP/(TP + FN). The recall is intuitively the ability of the classifier to find all the positive samples. The precision is the ratio TP/(TP + FP). The precision is intuitively the ability of the classifier not to label as positive a sample that is negative. The accuracy is the ratio (TP + TN)/(TP + FP + TN + FN). The accuracy is intuitively the ability of the classifier to label a sample that strictly matches with the reality. Figure 5 shows the development of the ML module, consisting of a data pre-processing part and a model construction part. The data pre-processing part included:

- Data cleaning: inconsistencies in the data (e.g., in the naming of batches) were corrected.
- Feature design: the output variable (i.e., whether the concentration of AFB1 in a feed batch was above or below the EC legal limit of 0.02 mg/kg) was added manually.
- Variable selection: Sampling month, feed product, feed product subgroup, feed product group, country of origin, and country of analysis were selected as input variables for ML.

The model construction part included:

- Data split: the entire data set spanning 2005–2015 was split randomly into training (90% of all data) and testing (10%) data subsets used for internal validation. Data of 2016–2018 were used for external validation.
- Model training and internal validation: the models’ parameters were tuned to improve the models’ performance on the training dataset using cross-validation (CV). Test data were used as internal validation for testing the model performance. The model was run using the default setting of these parameters, and then re-run using the tuned parameters. Because the data were highly unbalanced, adjusting the parameters is necessary to get a relatively better model performance. The model with the best performance, using the four above-mentioned evaluation metrics (accuracy, AUC, recall, and precision), was selected to design the monitoring plan.
- External validation: the 2016–2018 dataset was used for external validation. Feature importance was calculated, providing a score that indicates how valuable each feature was in the construction of the model^35^.

### Economic module

To estimate the cost of the monitoring program, the monitoring cost was calculated based on the predicted results of the feed batches (TP, FP, TN or FN), and the costs of respective follow-up actions (Table 6). These follow-up actions were: a) accept batch, b) store batch for further sampling and analysis (S&A) (as prescribed by EU Regulation No. 401/2006, including the number of samples collected from one batch, the analytical method used, and the acceptance and rejection rules), or c) recall and destroy the unused contaminated batch, and estimate the impact of already used contaminated batch on human health. The costs related to the different follow-up actions are presented in Table 7. The input parameter values related to costs were collected based on scientific literature, open data and online news sources.

**Table 6:** Monitoring cost related to the classification decision of the ML model, and used as one of the criteria for ML selection.

Prediction result	Definition of the prediction result	Monitoring		
		Follow-up actions	Costs components per batch^a^	Explanation of the follow-up actions
True Positive	Non-compliant batch predicted to be non-compliant	Sampling & Analysis	C_coll_ + C_analy_ + C_stor_	Sampling & Analysis and holding, followed by rejection of the non-compliant feed batch to feed material traders
		Storage		
False Positive	Compliant batch predicted to be non-compliant	Sampling & Analysis	C_coll_ + C_analy_ + C_stor_	Sampling & Analysis and holding, followed by acceptance of the compliant batch
		Storage		
True Negative	Compliant batch predicted to be compliant	Accept	0	Accept compliant batch
False Negative	Non-compliant batch predicted to be compliant	Accept, Recall, Destroy, Replace, Estimate disease burden	P_recall_ * (C_recall_+ C_destr_+ C_price_)*10+(1 − P_recall_)* C_burden_	1. Accept based on prediction, or 2. Recall and other actions due to non-compliant feed batch that is used to produce compound feed and found to be contaminated later

^a^See Table 7 for explanation of the cost component variables.

**Table 7:** Included cost items in the monitoring cost calculation.

Description of cost items	Variable	Value	Unit	Reference
Labor costs of collecting 100 samples per batch^a^	C_colle_	1000	euro/batch	^27^
Costs of analysing one aggregate sample from one batch^b^	C_analy_	100	euro/batch	^27^
Costs of storage	C_stor_	96	euro/batch	^27^
Costs of recall	C_recall_	5800	euro/batch	^28^
Percentage feed recalled	P_recall_	60	%	^28^
Costs of destruction	C_destr_	500	euro/batch	^28^
Costs of replacement	C_price_	22000	euro/batch	^28^
Costs of disease burden	C_burden_	30987	euro/batch	^26^

^a^Here we assumed 100 tonnes per feed batch; 100 incremental samples were collected per batch and combined into one aggregate sample.

^b^One aliquot was extracted from the aggregate sample to determine the AFB1 concentration using liquid chromatography combined with mass spectrometry (LC-MS/MS) with a detection limit of 0.0125 µg/kg.

#### Cost of the designed monitoring program

Table 6 presents the different follow-up actions following the prediction result. Batches predicted to be non-compliant (TP and FP) were collected and analyzed (S&A) to verify the contamination status; *C*_*colle*_ and *C*_*analy*_ represented the costs for, respectively, sampling and AFB1 analyses per tonne of feed material. Pending the monitoring results, batches were stored at a cost (*C*_*stor*_) of 0.96 €/t per day. The holding time was assumed to be two days. If the batch was found to be non-compliant (TP), the feed factories rejected the batch and returned it to the feed material trader, with no transport costs for the feed factory. Instead, the cost for transport and delivery of a new batch were for the trader. If predicted to be compliant (FP), the feed factories accepted this batch.

The total cost related to one TP or FP estimated batch was calculated by the following equations:

1 $$TP_{cost} = C_{colle} + C_{analy} + 2 \times C_{stor}$$

2 $$FP_{cost} = C_{colle} + C_{analy} + 2 \times C_{stor}$$

Batches predicted as compliant (TN and FN) were accepted by the feed factories to produce compound feed, without undergoing AFB1 testing (no S&A or storage costs). TN batches did not lead to any cost since there was no S&A or contamination of compound feed. FN batches, however, led to a recall and other costs. These FN batches, which were initially assumed to be compliant, were assumed to be found to be AFB1 contaminated in the compound feed in the consecutive downstream stage of the chain (dairy farm) by other organizations, resulting into recall and related costs, or cost-related to disease burden. We assumed one FN batch of a certain raw material (100 tonnes) was used to produce one batch of compound feed (1000 tonnes), resulting in contamination of 10% of compound feed with a concentration of AFB1 of 20 µg/kg. It was assumed that other organizations conduct regular S&A at the second stage of the supply chain (from feed factories to dairy farm); the cost of the regular checks are not considered by the feed factories; the contaminated compound feed was 100% detected, which is an extreme condition. Any unused contaminated compound feed at the dairy farm was directly recalled, destroyed, and replaced. The already used contaminated compound feed by the dairy farms resulted into disease burden cost due to human health effects of AFM1 presence in dairy products. *P*_*recall*_ represented the recall percentage of contaminated compound feed—caused by one FN batch that had not been used in the next stage of the chain, i.e., had not yet been consumed by dairy cows. The costs included: *C*_*recall*_ the per batch cost to recall the contaminated compound feed; *C*_*destr*_ the per batch cost to destroy it, and *C*_*price*_ the per batch cost to replace it. 1−*P*_*recall*_ represented the percentage of consumed contaminated compound feed, from one FN batch. This led to disease burden costs (*C*_*burden*_), per FN batch, related to the impact on human health. It was assumed that contaminated compound feed produced from the FN batch. We assumed a 100,000 population who consumed dairy products from this dairy supply chain during a period of one year. The resulting human disease burden was expressed as disability-adjusted life years (DALYs), indicating the loss of quality and quantity of life due to exposure to AFB1 via dairy consumption. DALYs caused by one FN batch (20 μg/kg AFB1 and 10% contamination) were calculated as 0.313 DALYs/100,000 population using the model designed by Wang, et al.^20^. The estimated cost for one DALY was 99,000 EUR derived from RIVM (National Institute for Public Health and the Environment https://www.rivm.nl/publicaties/disease-burden-of-food-related-pathogens-in-netherlands-2018). Cburden caused by one FN batch was thus calculated as 0.313 DALY * 99,000 Euro, equaling 30,987 EUR. The monitoring cost for one FN batch was calculated using the following equation:

3 $$FN_{cost} = P_{recall} \times \left( {C_{recall}} \right. + C_{destr} + \left. {C_{price}} \right) \times 10 + \left( 1 \right. - \left. {P_{recall}} \right) \times C_{burden}$$

The total cost of the monitoring program for AFB1 in feed was estimated by the following equation:

4 $$TC_{desi} = N_{FN} \times FN_{cost} + N_{TP} \times TP_{cost} + N_{FP} \times FP_{cost}$$

Where N_FN_, N_TP_, and N_FP_ were the number of batches predicted to be FN, TP, and FP, respectively.

#### Cost of current official monitoring

Available monitoring data represented test results of S&A in the period 2005–2018. Most of the samples were collected following Regulation (EC) No 401/2006. We assumed these data represented the results of a national official monitoring program. The total cost of the current official monitoring for AFB1 in feed was calculated by the following equation:

5 $$TC_{{{{\mathrm{curr}}}}} = \left( {N_{{{{\mathrm{FN}}}}} + N_{{{{\mathrm{TP}}}}} + N_{{{{\mathrm{FP}}}}} + N_{{{{\mathrm{TN}}}}}} \right) \times \left( {C_{colle} + C_{analy}} \right)$$

The percentage of cost reduction of the designed monitoring program compared to the current official monitoring for AFB1 in feed was calculated by the following equation:

6 $$Cost\_reduction = \left( {TC_{{{{\mathrm{curr}}}}} - TC_{desi}} \right)/TC_{{{{\mathrm{curr}}}}}$$

## Data Availability

Data used are from the official control program of food safety hazards in animal feed in the Netherlands (via project WOT-02-004-012) and cannot be shared.

## Code availability

Code for data analysis is available at https://github.com/manman1010/monitoring.git.
