# Author Correction: Designing a monitoring program for aflatoxin B1 in feed products using machine learning

**DOI:** 10.1038/s41538-022-00160-4

**Published:** 2022-09-20

**Authors:** X. Wang, Y. Bouzembrak, A. G. J. M. Oude Lansink, H. J. van der Fels-Klerx

**Affiliations:** 1 Business Economics, Wageningen University, Wageningen, The Netherlands; 2 Wageningen Food Safety Research, Wageningen, The Netherlands

**Keywords:** Economics, Chemical safety

Correction to: *npj Science of Food* 10.1038/s41538-022-00154-2, published online 01 September 2022

In this article the affiliation ‘2Wageningen Food Safety Research, Wageningen, The Netherlands.’ for H. J. van der Fels-Klerx was missing. The panel legend for Figs. 1 and 2 inadvertently swapped. The original article has been corrected.

